# In memory of Professor KL Goh: The quintessential academic leader

**DOI:** 10.1002/jgh3.12945

**Published:** 2023-07-24

**Authors:** Ida Hilmi, Choon Jin Ooi, Vineet Ahuja, Rakesh Aggarwal

**Affiliations:** ^1^ Department of Medicine, Division of Gastroenterology and Hepatology, Faculty of Medicine University Malaya Kuala Lumpur Malaysia; ^2^ Asian Education Network in Inflammatory Bowel Disease; ^3^ Duke‐NUS Medical School Singapore; ^4^ Department of Gastroenterology All India Institute of Medical Sciences New Delhi India; ^5^ Department of Gastroenterology Jawaharlal Institute of Postgraduate Medical Education and Research Puducherry India

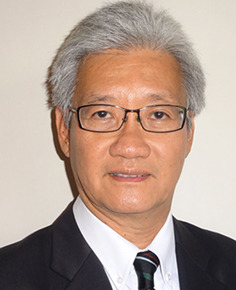

## My teacher and mentor

I joined Professor Khean Lee Goh's unit in 2003 when “I was a mere schoolgirl” as he liked to point out (I was 30 years old). At the time, I did not realize how privileged I was and how important a role he would play in my career. In the many of the tributes dedicated to him, there is a long list of the accolades he received. In this one, I wanted to highlight the things that were not written, that only some of us had the privilege to know…

KL Goh had an inimitable persona. He was a born leader and people gravitated toward him. His passion for teaching was legendary and his unit has trained almost a quarter of Malaysia's gastroenterologists, not to mention surgeons and fellows who came from abroad. He was a visionary. It is no coincidence that he established one the earliest endoscopy units at University Malaya Hospital in Kuala Lumpur and introduced the first international live endoscopy workshop 30 years ago. In recognition, the GI Endoscopy unit was honored with the prestigious “Centre of Excellence” award by the World Digestive Endoscopy Organization in 2008, a position it holds until today. It was only fitting that we inaugurated an oration for this year's Endoscopy Workshop Khean Lee Goh Distinguished Lecture, which we were very grateful that he was able to attend in person.

He was an avid researcher. He received the Merdeka Award in Medicine (a national award to celebrate the leaders in various academic fields) for his work on *Helicobacter pylori*. In addition, he contributed extensively to almost all fields related to gastroenterology and hepatology in Malaysia. He was a founder of the Malaysian Society of Gastroenterology and Hepatology, which evolved into a large organization hosting high‐quality scientific programs. Professor KL Goh was the congress president for APDW 2010, the first APDW congress held in Malaysia.

He had incredible work ethics, and one of my earliest memories at work was him trying to convince me the “joy” of waking up at 4 am every morning to write papers (I have to confess he did not achieve this aim). Although he was a senior consultant, he was there at almost every endoscopy list and the last to leave the clinic in spite of his heavy administrative duties and academic work. He was a classic case of leading by example. His fellows and staff adored him. He loved to teach medical students, for which he called it our “sacred duty.”

There are so many things I want to share—his deadpan humor, how he loved to tease his staff and gave them pseudo English names (George, Vincent, Rory to name a few…I was renamed Maria after watching the “Sound of Music”), his quirky love for history and travel. He had an amazing memory for names and faces, not just professionals but also everyone in the biomedical industry, hospital staff at every level, and almost everyone he met.

The last few years of illness were hard for us to watch but he is at peace now. We will fondly remember Professor Goh and his enduring legacy will always prevail…

Rest in peace, Professor, my mentor, father figure, inspiration, and friend……

‐*Ida Hilmi*

## The academic leadership

Live neither in the past nor in the future, but let each day's work absorb your entire energies, and satisfy your widest ambition. (Sir William Osler. *Aequanimitas ‘After 25 years.’* 1914; 213)

I have the wonderful privilege and honor to have known Professor KL Goh for the past 20 years. He was an eminent clinician, educator, leader, and mentor. In all my interactions with him, I have observed his unwavering dedication to his work and tenacity to finish a task no matter how challenging it may be. There was never an idle moment in his life, which he filled with his care for his patients and purpose in nurturing the younger generation of medical professionals. He would mentor, advise, and often offer a listening ear. Occasionally I see the weariness in his eyes, but his fortitude always carries him through. It is with this vivid memory that I choose to start this dedication with Sir William Osler's quotation. Professor Goh has amply lived the life espoused by Sir William Osler.

Throughout my medical career, from when I was a young registrar to consultant in the field of gastroenterology, he was always available to offer advice, counsel and friendship each and every time we meet in regional meetings. When he found out about my interest in inflammatory bowel disease (IBD), he supported my regional colleagues and I who were budding “IBD‐ologists” in pioneering IBD education and Asian guidelines. He advocated for the initiation of Clinical IBD Forums, organized under the auspices of Asian Pacific Association of Gastroenterology (APAGE). Leveraging on his constant support and enthusiasm, we have since successfully held four meetings in Penang (2014), Chiang Mai (2016), Cebu (2018), and Delhi (2022), respectively.

Professor Goh served as President of APAGE and Asian Pacific Digestive Week Foundation (APDWF) from 2010–2014 and 2014–2018, respectively. It was with his contributions during his tenure that these organizations are now synonymous with excellence in gastroenterology education.

In his busy academic life, he was a highly sought‐after speaker. At the WGO Distinguished Global Lecture APDW2015 in Taipei, he dealt with “Emerging GI and Liver Diseases in the Asia‐Pacific—Implications for Healthcare in the Region.” The *JGHF* Marshall & Warren Lecture on “Lessons Learnt from the Epidemiology of Helicobacter Pylori Infection in a Multiracial Asian Population in Malaysia” was delivered at APDW2017 in Hong Kong.

His exemplary leadership unified followers and skeptics alike who join him in the mission of advancing regional education and research, two key areas closest to his heart. My work with him at APAGE was an enriching experience. I learnt a great deal from this truly remarkable gentleman, the virtues of diplomacy, patience, fortitude, and empathy.

Professor KL Goh, we will miss you terribly.

‐*Choon Jin Ooi*

## Labor of love

Professor Goh was an extraordinary academician and his contributions to the scholarly community have been inspiring and immense. His hard work and contributions to the academic community can by gauged by his formidable Google Scholar *H* Index of 81. He worked on issues that were very relevant for Asia pacific region and in that process it was very natural that when Wiley planned a new journal *JGH Open*, they needed an academician not only with impeccable credentials but also someone who had friends across the globe and no one else could have been better than Professor Goh. And his acquiescence to be the Editor in Chief for the newly launched *JGH Open* was a blessing for the journal. *JGH Open*, which started in 2017 with just 4 issues a year, moved very fast up the ladder to be a journal with 12 issues a year and that too within a period of 4 years. This was testament to the ability of Professor Goh to maintain a very fine balance between inclusiveness as well as the ability to select high‐quality articles, his guidance in shaping content, and his commitment to maintain rigorous standards. *JGH Open* was like a sapling, which required constant and absolute attention to detail if it was to survive in extreme competitive environment of medical journals. His dedication to the journal was exemplary and that paved the way for the growth of *JGH Open*. It was an everyday effort that we associate editors could feel and it was in the form of the promptness in handling submissions, responsiveness to authors and reviewers, and the overall commitment to the publication process. He often went above and beyond to provide constructive feedback or mentorship to authors.

He had a marvelous way of leading the team of associate editors and the publishers, always very courteous, mindful of each one's time, trying to strike a subtle equilibrium between the aspirations of the publishers and the ambitions of the editorial team and ensuring that everyone enjoyed working with him. He was a gentleman to the core, extremely gracious and yet fully committed at the same time. Such a combination of extraordinary attributes is hard to get and that is what *JGH Open* got at its helm. No wonder that when impact factors were announced last week, *JGH Open* got its first impact factor (IF: 1.7), which is a wonderful start. As Sanjiv Mahadeva wrote on hearing the news of *JGH Open* getting an opening IF “A lot of this has been due to the efforts of the late Prof KL Goh, founding Editor‐in‐chief and we will always be grateful for his vision. I only wish he could have seen the fruition of his labour of love.”

‐*Vineet Ahuja*

## And a leader for the Asia‐Pacific gastroenterology community

My first contact with Professor KL Goh was quite late in life, in 2013, when I joined the *Journal of Gastroenterology and Hepatology Foundation*, of which he was the Chair at that time.

Professor KL Goh served the *Journal of Gastroenterology and Hepatology Foundation* (https://www.jghfoundation.org.au) as a Trustee from 2002 to 2016, including the last four years as its Chair. Professor Goh was a great leader, with an inimitable style characterized by a gentle and understated demeanor. He was deeply interested in fostering education and research in the field of gastrointestinal sciences in the Asia‐Pacific medical community. He embodied collegiality, welcoming all and creating links between clinicians in the region who could further research and improvements in GI care. He recognized appreciatively the contribution of all those involved in work of the Foundation, and passed on the credit to others for work that had in fact been done by him behind the scene.

During his time with the *JGHF*, he was involved in establishing a number of awards and supporting a range of projects around the Asia‐Pacific region. I vividly recall the meeting where I first proposed starting of a Young Clinician Investigator Programme as a one‐day pre‐conference activity at the annual APDW meetings to teach the young participants, mostly trainees, the basics of research methodology. He immediately supported the idea though it meant a significant expense on part of the Foundation, and saw to it that it was implemented. He made sure to take time out to attend some of the sessions at the first iteration, and to provide his feedback.

He was deeply interested in the progress of the *Journal of Gastroenterology and Hepatology*, which he sincerely believed in as providing a forum to gastroenterologists in the Asia‐Pacific region for publishing their work. He was always open to new ideas and experimentation. Thus, with the advent of the author‐pay model for publication and realizing the need for this for the Asia‐Pacific gastroenterology community, he worked closely with Wiley to help set up the *JGH Open*, an open‐access journal. He decided to give up his position as Chair of the Foundation ahead of the scheduled time, to work toward the success of this journal and give it his undivided attention. He nurtured the *JGH Open* as a parent does a baby. He regularly implored the Foundation for support to it through funding the publication fees for articles submitted to it for the initial few years. This, we believe, was one of the steps that paved the way for the eventual success of this endeavor.

My last meeting with him was in late 2022 in Kuala Lumpur, when the Foundation decided to felicitate him for his contributions. He was his usual self, sharing several thoughts to further the causes that he believed in.

His gentle nature, kindness, and humor will be missed by everyone who knew him, including all of us at the Foundation.

‐*Rakesh Aggarwal*

